# Effects of body mass index and serum albumin on overall survival in patients with cancer undergoing pancreaticoduodenectomy: a single-center retrospective cohort study

**DOI:** 10.1186/s12957-022-02678-z

**Published:** 2022-07-01

**Authors:** Lei Cui, Huiping Yu, Qingmei Sun, Yi Miao, Kuirong Jiang, Xiaoping Fang

**Affiliations:** Department of General Surgery, Pancreas Center, The First Affiliated Hospital with Nanjing Medical University, Nanjing, China

**Keywords:** Albumin, Body mass index, Pancreaticoduodenectomy, Pancreatic cancer, Periampullary carcinoma, Survival

## Abstract

**Objectives:**

We aimed to explore whether body mass index (BMI) and albumin were associated with overall survival (OS) in individuals who underwent pancreaticoduodenectomy (PD) for cancer.

**Methods:**

Three-hundred twenty-nine consecutive patients who underwent PD for cancer were enrolled from January 2020 to December 2020. All clinicopathological information was extracted based on medical records. The survival follow-ups were regularly performed and ended on June 30, 2021. The Kaplan-Meier survival analysis and univariate and multivariate Cox proportional-hazards models were used to assess the association of BMI and albumin with OS.

**Results:**

Of the 329 patients, 186 (56.5%) were male, and median age at admission was 65.0 (56.0–71.0) years. There were 258 patients (78.4%) with *BMI* < 25.0 kg/m^2^ and 89 patients (27.05%) with albumin < 35.0 g/L respectively. In overall cohort, *BMI* < 25.0 kg/m^2^ was associated with OS (adjusted *HR* = 3.516, 95% *CI* = 1.076–11.492, *P* = 0.037). In contrast, albumin < 35.0 g/L did not affect OS. Subgroup analysis showed, in patients with pancreas lesion, *BMI* < 25.0 kg/m^2^ had a higher risk for OS compared to BMI ≥ 25.0 kg/m^2^ (adjusted *HR* = 3.209, 95% *CI* = 0.985–10.451, *P* = 0.048), while albumin < 35.0 g/L was not linked to OS. In patients with lesion in ampulla of Vater, duodenum, or common bile duct, there was no significant association of BMI and albumin with OS.

**Conclusions:**

BMI, rather than serum albumin, was associated with OS in patients who underwent PD for cancer.

**Supplementary Information:**

The online version contains supplementary material available at 10.1186/s12957-022-02678-z.

## Introduction

Since the middle of twentieth century, pancreaticoduodenectomy (PD) has been considered as a primary treatment strategy for lesion regions located in the head of pancreas and periampullary [1]. Recently, advances in surgical techniques combined with evidence-based perioperative management have improved the safety and reliability of PD procedure, but long-term overall survival (OS) remains unacceptably considerable because of the complexity of surgical procedure and the characteristic of lesion [2, 3]. A large sample study reported that 1-, 3-, 5-, and 10-year mortality rates were 15%, 65%, 85%, and 93%, respectively [3], and even these value may be higher in some Pancreas Center. Identifying the predictors of mortality after PD and assigning corresponding measures can help improve long-term survival outcomes.

Malnutrition occurs frequently in patients before pancreatic surgery, and its prevalence fluctuates from 36 to 88% according to different diagnostic criteria [4]. As an intervenable factor, nutritional status has been used to explore the effects on postoperative short-term and long-term outcomes in patients who underwent major oncologic surgery [5–16]. Despite validated nutritional assessment tools are various (such as weight loss [17], muscle mass [18], Mini Nutrition Assessment (MNA) scores [19], and subjective global assessment (SGA) scores [20]), body mass index (BMI) [21] and albumin [22] are still the most widely utilized due to their easy availability. Although there were several studies investigating the association of BMI and albumin with OS in patients after PD, their findings were under debate and could not represent the characteristic of Chinese population [23–26]. There were two studies investigating the relationships between BMI and OS in patients undergoing PD [23, 24]. A study in USA found that, compared with normal weight patients (*BMI* < 25 kg/m^2^), obese patients (*BMI* > 30 kg/m^2^) who underwent PD for pancreatic cancer had an improved long-term survival independent of known clinicopathologic factors [23]. However, the authors excluded underweight patients (*BMI* < 18.5 kg/m^2^) from normal weight cohort, which limits statistical power because *BMI* < 18.5 kg/m^2^ are considered to be a known risk factor for OS in patients who followed major oncologic intra-abdominal surgery [27]. The other study by Chang et al. showed that obesity (*BMI* > 30 kg/m^2^) increased risks of postoperative 30-day mortality in patients after PD [24]. However, that study lacked long-term follow-up data. The following two studies explore whether albumin is a prognostic factor of OS in patients undergoing PD [25, 26]. A study by Şeren et al. presented that albumin levels < 25 g/L were an independent risk for OS in periampullary cancer patients who underwent PD [26]. However, they only enrolled 89 participants. The other large sample study demonstrated preoperative albumin < 35 g/L increased risks of mortality in patients undergoing PD [25]. However, that study only enrolled patients with resectable pancreatic tumor anatomy but marginal performance status or reversible comorbidities. As a result, whether preoperative BMI or serum albumin considered as long-term prognostic factors for OS in individuals receiving PD for cancer remains unclear.

Therefore, this large-sample retrospective survey was conducted to collect clinicopathological information and 18-month follow-up data of patients who underwent PD due to malignant lesions in the pancreas, ampulla of Vater, duodenum, or common bile duct, which aimed to address whether preoperative BMI and serum albumin were prognostic factors of long-term OS in patients undergoing PD.

## Methods

### Study design

From January 2020 to December 2020, a consecutive series of 453 patients who underwent elective PD in Pancreas Center of the First Affiliated Hospital of Nanjing Medical University (Nanjing, China) were evaluated. Patients were included in this study if they meet the following criteria: (1) aged 18 years and older and (2) pathologically diagnosed as malignant tumor. Exclusion criteria were as follows: (1) patients who had performed other abdominal surgery, (2) patients with liver disease, (3) patients who had missing data of preoperative BMI or albumin, and (4) patients with more than grade 3 of the American Society of Anesthesiologists (ASA) classification. This study was performed based on the ethical standards of the Helsinki Declaration. Due to the retrospective nature of this study, participants’ informed consent was waived.

### Surgical procedures

When performing the PD surgery, it was determined whether to resect the pylorus according to the indication. If the tumor invaded the pylorus or duodenum, the pylorus-resecting PD was conducted, or else, the pylorus-preserving PD was performed. An extended removal was taken into consideration to reach negative margin if the tumors were growth in tissues beyond the standard resection range, involving in portal vein or superior mesenteric vein, transverse mesenteric vessels, whole stomach, and segmental colon. Reconstruction of the digestive tract was carried out by cholangiojejunostomy, pancreaticojejunostomy, and gastrojejunostomy.

### Data collection

All data were extracted based on clinical electronic medical record. Following preoperative clinical data, age, gender, BMI, history of smoking and alcohol drinking, coexisting diseases (i.e., diabetes, hypertension, and coronary heart disease), and ASA score were collected. Laboratory parameters involving in serum albumin levels, alanine transaminase (ALT), C-reactive protein (CRP), total bilirubin, carbohydrate antigen 19-9 (CA-199), and carcinoembryonic antigen (CEA) were measured within 3 days before surgery. Intraoperative data containing operative lasting time, estimated blood loss, the presence of blood transfusion, type of procedures, pathological information, and lesion location were collected. Postoperative data were collected as follows: surgery-related complications, hospital length of stay, postoperative stay length, reoperation, and 90-day readmission. All individuals were followed up until June 30, 2021.

### Definition of parameters

BMI was calculated by the weight in kilograms divided by the height in meters squared. All of patients were divided into group 1 and group 2 according to whether *BMI* < 25 kg/m^2^ or BMI ≥ 25 kg/m^2^ [28]. And participants were divided into two groups (group I and group II) based on whether the serum albumin levels were lower than 35 g/L [29]. OS was defined as any death from surgery to the end of follow-up. Postoperative complications were graded based on the Clavien–Dindo classification [30]. Major complications were determined by a grade of Clavien–Dindo classification exceeding 2 [31]. Hemorrhage [32], pancreatic fistula (PF) [33], and delayed gastric emptying (DGE) [34] were diagnosed based on the International Study Group of Pancreatic Surgery (ISGPS). Biliary leakage was defined as the following criteria: bile fluid in the abdominal drainage or radiographically confirmed effusion requiring percutaneous drainage and showing elevated bilirubin levels [35]. A diagnosis of chyle leakage was considered if meeting the following criteria: milk-like fluid was drawn from the drainage tube or wound with the triacylglycerol concentration exceeding 110 mg/dL after postoperative 3 days [36]. The diagnosis of intra-abdominal infection was based on confirmed intra-abdominal fluid by ultrasound or computed tomography, while in patients with additional persistent fever and white blood cell elevation, signs of infection by radiological examination or positive bacteria culture from collected fluid [37].

### Statistical analysis

Categorical variables were presented as number and proportion, and their differences between groups were analyzed using the method of person chi-square test or Fisher’s exact test. Depending on whether they showed normal distribution or not, continuous variable were described as mean with standard deviation or median with interquartile range, and differences between groups were analyzed by independent *t*-test or Mann-Whitney *U*-test. Cumulative OS were calculated through the Kaplan–Meier method, and differences between curves were analyzed using the log-rank test. The effects of clinicopathological factors on OS were evaluated using the univariable Cox proportional-hazards model. Any variable where *P* < 0.1 in univariable analysis was regarded a candidate for multivariable analysis. Hazard ratios (HR) and 95% confidence intervals (CI) were provided. Statistical analyses were performed using the software package of IBM SPSS Statistics for Windows version 25.0 (SPSS Inc.). *P* < 0.05 was considered statistically significant (two-tailed test).

## Results

### Enrolled patients

In this study, 124 individuals were ineligible, including 15 with history of other abdominal operations (such as colectomy or gastrectomy), 7 with comorbidity of liver disease, 2 with missing data of BMI or preoperative albumin, 96 with benign lesions, and 4 with exceeding grade 3 of the American Society of Anesthesiologists (ASA) classification. Finally, a total of 329 participants undergoing PD were enrolled in this retrospectively cohort study.

### Patient characteristics

The baseline characteristics of participants were presented in Table 1. Among 329 patients, 56.5% of them were male, and median age at admission was 65.0 (56.0–71.0) years. Of the 329 participants, 258 patients (78.4%) were with *BMI* < 25.0 kg/m^2^, and 89 patients (27.05%) were with serum albumin < 35.0 g/L. There were no significant differences in age, sex, smoking history, alcohol history, ASA class, the presence of diabetes, the presence of hypertension, the presence of coronary heart disease, cancer stage, lesion location, HbA1c, ALT, CRP, CEA, CA-199, total albumin, and total bilirubin between two groups of patients with *BMI* < 25.0 kg/m^2^ and BMI ≥ 25.0 kg/m^2^. Patients with albumin < 35.0 g/L had significantly higher age, CRP, CA-199, and total bilirubin than those with albumin ≥ 35.0 g/L. Also, proportion of ASA class, cancer stage, and lesion location was observed significantly in differences between two groups of patients with albumin < 35.0 g/L and albumin ≥ 35.0 g/L. But significant differences were not observed in age, smoking history, alcohol history, the presence of diabetes, HbA1c, the presence of hypertension, the presence of coronary heart disease, ALT, CEA, and BMI between patients with albumin < 35.0 g/L and albumin ≥ 35.0 g/L.

**Table 1:** Baseline characteristics of study participants (*n* = 329)

Variables	Total (***n*** = 329)	Group 1 (***n*** = 258)	Group 2 (***n*** = 71)	***P-***value	Group I (***n*** = 89)	Group II (***n*** = 240)	***P***-value
Age (years)	65.0 (56.0–71.0)	65.0 (57.0–71.0)	64.0 (54.0–69.0)	0.131	69.0 (61.5–75.5)	64.0 (56.0–69.0)	0.000
Sex (*n*/%)				0.297			0.356
Male	186 (56.5)	142 (55.0)	44 (62.0)		54 (60.7)	132 (55.0)	
Female	143 (43.5)	116 (45.0)	27 (38.0)		35 (39.3)	108 (45.0)	
Smoking history (*n*/%)	63 (19.1)	52 (20.2)	11 (15.5)	0.377	15 (16.9)	48 (20.0)	0.519
Alcohol history (*n*/%)	40 (12.2)	33 (12.8)	7 (9.9)	0.503	31 (12.9)	9 (10.1)	0.489
ASA class (*n*/%)				0.219			0.000
I	9 (2.7)	5 (1.9)	4 (5.6)		1 (1.1)	8 (3.3)	
II	259 (78.7)	206 (79.8)	53 (74.6)		58 (65.2)	201 (83.8)	
III	61 (18.5)	3 (18.2)	14 (19.7)		30 (33.7)	31 (12.9)	
Diabetes (*n*/%)	75 (22.8)	55 (21.3)	20 (28.2)	0.223	24 (27.0)	51 (21.3)	0.272
HbA1c (%)	5.8 (5.3–6.7)	5.7 (5.3–6.5)	6.2 (5.4–6.8)	0.106	5.8 (5.2–7.8)	5.8 (5.3–6.6)	0.618
Hypertension (*n*/%)	115 (35.0)	87 (33.7)	28 (39.4)	0.371	31 (34.8)	84 (35.0)	0.977
CHD (*n*/%)	8 (2.4)	4 (1.6)	4 (5.6)	0.070	2 (2.2)	6 (2.5)	1.000
ALT (U/L)	107.8 (31.0–243.6)	108.3 (28.4–253.5)	107.3 (38.4–233.2)	0.723	110.5 (48.6–172.0)	105.2 (22.0–275.6)	0.833
CRP (mg/L)	5.8 (2.8–13.8)	5.7 (2.8–12.9)	6.8 (2.9–15.3)	0.574	8.4 (5.5–26.1)	4.8 (2.4–11.8)	0.000
CEA (ng/ml)	3.2 (2.2–4.8)	3.3 (2.2–5.1)	3.1 (2.1–4.5)	0.313	3.4 (2.5–5.1)	3.2 (2.1–4.7)	0.279
CA-199 (U/ml)	115.6 (35.3–367.0)	122.1 (34.7–423.4)	100.5 (39.7–321.4)	0.601	168.9 (42.9–718.5)	108.6 (31.7–218.3)	0.022
BMI (kg/m^2^)	22.8 (20.8–24.8)	22.0 (20.3–23.4)	26.4 (25.6–27.3)	0.000	22.5 (20.5–24.4)	22.9 (21.0–24.8)	0.517
Albumin (g/L)	37.4 (34.5–40.5)	37.4 (34.4–40.2)	38.8 (34.5–41.3)	0.205	33.1 (30.7–33.9)	39.3 (37.0–41.8)	0.000
TB (mmol/L)	81.5 (15.0–200.7)	81.7 (14.8–200.1)	77.7 (16.3–201.9)	0.454	153.8 (58.9–288.5)	37.4 (12.8–165.1)	0.000
Cancer stage (*n*/%)				0.141			0.009
I	118 (35.9)	96 (37.2)	22 (31.0)		43 (48.3)	75 (31.3)	
II	161 (48.9)	128 (49.6)	33 (46.5)		32 (36.0)	129 (53.8)	
III	50 (15.2)	34 (13.2)	16 (22.5)		14 (15.7)	36 (15.0)	
Location (*n*/%)				0.076			0.009
Pancreas	229 (69.6)	181 (70.2)	48 (67.6)		50 (56.2)	179 (74.6)	
Ampulla of Vater	27 (8.2)	22 (8.5)	5 (7.0)		12 (13.5)	15 (6.3)	
Duodenum	34 (10.3)	30 (11.6)	4 (5.6)		14 (15.7)	20 (8.3)	
Common bile duct	39 (11.9)	25 (9.7)	14 (19.7)		13 (14.6)	26 (10.8)	

Group 1, *BMI* < 25 kg/m^2^; group 2, *BMI* ≥ 25 kg/m^2^; group I, serum albumin < 35 g/L; group II, serum albumin ≥ 35 g/L

*ALT* alanine transaminase, *ASA* American Society of Anesthesiologists, *BMI* body mass index, *CA-199* carbohydrate antigen 19-9, *CEA* carcinoembryonic antigen, *CHD* coronary heart disease, *CRP* C-reactive protein, *TB* total bilirubin

### Surgical outcomes

Table 2 showed the surgical outcomes of 329 eligible individuals. The median (interquartile range) of operative time, hospital stay, and postoperative length of stay was 255.0 (210.0–307.8) min, 19.0 (15.0–27.0) days, and 14.0 (10.0–21.0) days, respectively. There were 131 patients (39.8%) with major complications, 52 patients (15.8%) with DGE, 61 patients (18.5%) with PF, 7 patients (2.1%) with biliary leakage, 6 patients (1.8%) with chyle leakage, 16 patients (4.9%) with hemorrhage, 13 patients (4.0%) with wound infection, 41 patients (12.5%) with abdominal infection, 18 patients (5.5%) with readmission within 90 days, and 7 patients (2.1%) with reoperation, respectively. Between two groups of patients with *BMI* < 25.0 kg/m^2^ and *BMI* ≥ 25.0 kg/m^2^, no significant differences were observed in operation time, hospital length of days, postoperative hospital length of days, the presence of major complication, DGE, biliary leakage, chyle leakage, hemorrhage, wound infection, abdominal infection, 90-day readmission, and reoperation, while patients with *BMI* ≥ 25.0 kg/m^2^ had lower risks of PF compared to those with *BMI* < 25.0 kg/m^2^ (26.8% vs. 16.3%, *P* = 0.044). Between two groups of patients with albumin < 35.0 g/L and albumin ≥ 35.0 g/L, there were no significant differences in operation time, hospital length of days, postoperative hospital length of days, the presence of major complication, DGE, PF, biliary leakage, chyle leakage, wound infection, abdominal infection, 90-day readmission, and reoperation, while patients with albumin < 35.0 g/L had higher risks of hemorrhage than those with albumin ≥ 35.0 g/L (9.0% vs. 3.3%, *P* = 0.034).

**Table 2:** Surgical outcomes of study participants (*n* = 329)

Variables	Total (***n*** = 329)	Group 1 (***n*** = 258)	Group 2 (***n*** = 71)	***P-v***alue	Group I (***n*** = 89)	Group II (***n*** = 240)	***P-***value
Operation type (*n*/%)				0.933			0.753
Open	302 (91.8)	237 (91.9)	65 (91.5)		81 (91.0)	221 (92.1)	
Laparoscopy	27 (8.2)	21 (8.1)	6 (8.5)		8 (9.0)	19 (7.9)	
Surgical type (*n*/%)				0.935			0.076
PD	196 (59.6)	154 (59.7)	42 (59.2)		46 (51.7)	150 (62.5)	
PPPD	133 (40.4)	104 (40.3)	29 (40.8)		43 (48.3)	90 (37.5)	
Operation time (min)	255.0 (210.0–307.8)	245.0 (207.0–305.5)	270.0 (240.0–316.0)	0.028	245.0 (212.5–305.0)	255.0 (210.0–308.0)	0.686
Estimated blood loss (ml)	200.0 (200.0–400.0)	200.0 (200.0–400.0)	200.0 (150.0–400.0)	0.667	200.0 (162.5–300.0)	200.0 (200.0–400.0)	0.118
Transfusion (*n*/%)	68 (21.4)	53 (21.1)	15 (22.4)	0.821	19 (22.1)	49 (21.1)	0.851
Hospital LOS (days)	19.0 (15.0–27.0)	18.3 (14.0–27.0)	20.0 (15.5–29.0)	0.197	20.0 (15.0–28.0)	19.0 (14.3–27.0)	0.622
Postoperative LOS (days)	14.0 (10.0–21.0)	13.5 (10.0–20.3)	14.0 (11.0–25.0)	0.206	14.0 (10.0–21.0)	14.0 (10.0–21.0)	0.983
Major complication (*n*/%)	131 (39.8)	100 (38.8)	31 (43.7)	0.455	41 (46.1)	90 (37.5)	0.158
DGE (*n*/%)	52 (15.8)	41 (15.9)	11 (15.5)	0.935	16 (18.0)	36 (15.0)	0.511
Pancreatic fistula (*n*/%)	61 (18.5)	42 (16.3)	19 (26.8)	0.044	16 (18.0)	45 (18.8)	0.873
Biliary leakage (*n*/%)	7 (2.1)	5 (1.9)	2 (2.8)	0.647	8 (3.3)	8 (3.3)	8(3.3)
Chyle leakage (*n*/%)	6 (1.8)	5 (1.9)	1 (1.4)	1.000	1 (1.1)	5 (2.1)	1.000
Hemorrhage (*n*/%)	16 (4.9)	12 (4.7)	4 (5.6)	0.756	8 (9.0)	8 (3.3)	0.034
Wound infection (*n*/%)	13 (4.0)	12 (4.7)	1 (1.4)	0.313	4 (4.5)	9 (3.8)	0.754
Abdominal infection (*n*/%)	41 (12.5)	31 (12.0)	10 (14.1)	0.640	11 (12.4)	30 (12.5)	0.973
90-day readmission (*n*/%)	18 (5.5)	13 (5.0)	5 (7.1)	0.553	7 (7.9)	11 (4.6)	0.277
Reoperation (*n*/%)	7 (2.1)	7 (2.7)	0 (0.0)	0.353	3 (3.4)	4 (1.7)	0.393

Group 1, *BMI* < 25 kg/m^2^; group 2 ≥ 25 kg/m^2^; group I, serum albumin < 35 g/L; group II, serum albumin ≥ 35 g/L

*DGE* delayed gastric emptying, *LOS* length of stay, *PD* pancreaticoduodenectomy, *PPPD* pylorus-preserving pancreaticoduodenectomy

### Survival analysis of all cohorts

**Fig. 1:**
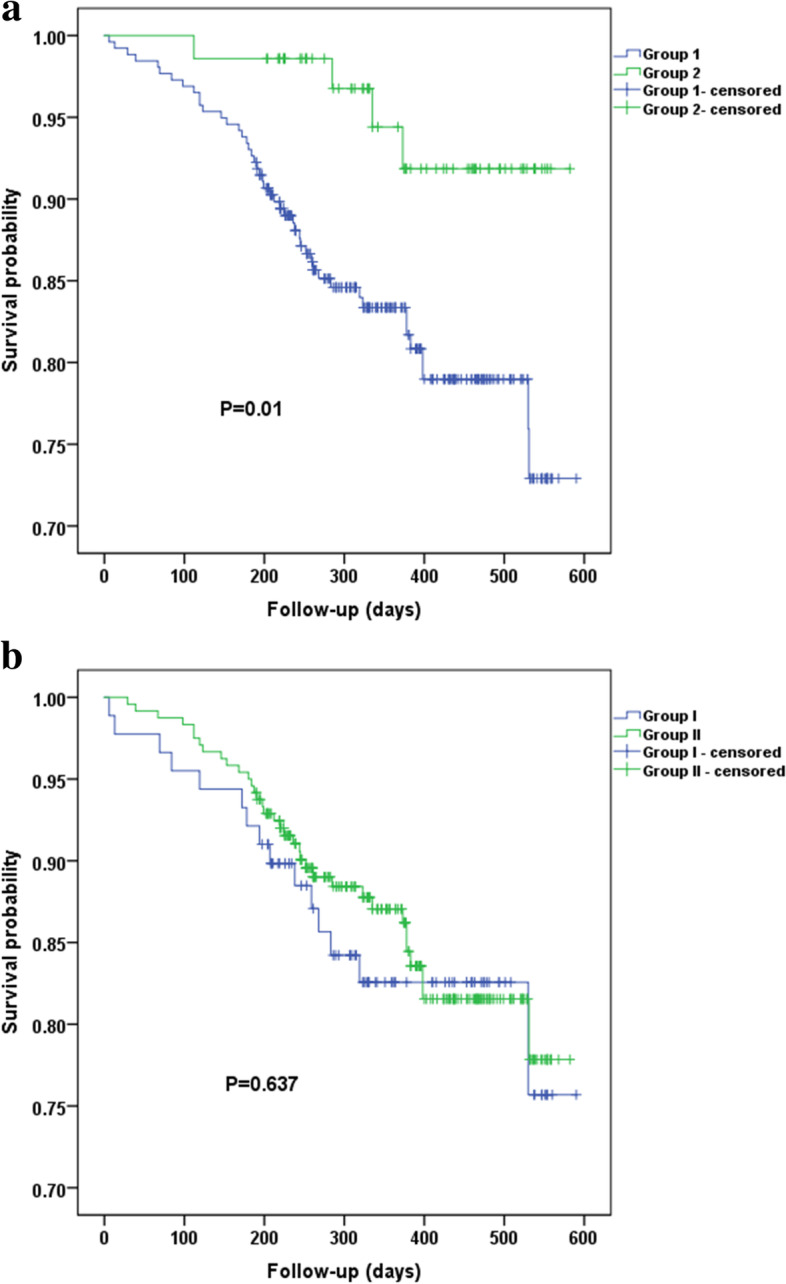
**a** Kaplan-Meier survival curves for overall cohort according to BMI (< 25.0 kg/m^2^ vs. ≥ 25.0 kg/m^2^). (Note: Group 1, *BMI* < 25 kg/m^2^; group 2, ≥ 25 kg/m^2^). **b** Kaplan-Meier survival curves for overall cohort according to albumin (< 35.0 g/L vs. ≥ 35.0 g/L). (Note: Group I, serum albumin < 35 g/L; group II, serum albumin ≥ 35 g/L)

In overall cohort, univariate analysis showed that *BMI* < 25.0 kg/m^2^ was significantly linked to OS (Fig. 1a), while albumin < 35.0 g/L was not (Fig. 1b). Further multivariate analysis presented that *BMI* < 25.0 kg/m^2^ (*HR* = 3.516, 95% *CI* = 1.076–11.492, *P* = 0.037) remained independent prognostic factors after adjusting age, CRP, cancer stage, and lesion locations (Table 3).

**Table 3:** Univariate and multivariate analysis of factors associated with overall survival (*n* = 329)

Variables	Unadjusted HR (95% ***CI***)	***P-***value	Adjusted HR (95% ***CI***)	***P-***value
Age (≥ 65 years) (*n*/%)	2.610 (1.407–4.841)	0.002	2.452 (1.294–4.647)	0.006
Male (*n*/%)	1.074 (0.613–1.884)	0.802		
Smoking history (*n*/%)	0.731 (0.343–1.559)	0.418		
Drinking history (*n*/%)	0.408 (0.127–1.312)	0.132		
Diabetes (*n*/%)	1.086 (0.568–2.079)	0.803		
Hypertension (*n*/%)	1.050 (0.589–1.871)	0.868		
CHD (*n*/%)	0.048 (0.000–240.451)	0.485		
ASA class (II)	1.409 (0.193–10.280)	0.735		
ASA class (III)	2.337 (0.306–17.872)	0.414		
BMI < 25.0 kg/m^2^	3.533 (1.271–9.816)	0.015	3.516 (1.076–11.492)	0.037
CA-199 (U/ml)	1.000 (1.000–1.001)	0.202		
CEA (ng/ml)	0.965 (0.900–1.034)	0.309		
CRP (mg/L)	1.009 (1.000–1.018)	0.063	1.001 (1.002–1.020)	0.012
HbA1c (%)	0.911 (0.713–1.165)	0.458		
ALT (U/L)	1.000 (0.999–1.002)	0.822		
TB (μmol/L)	1.000 (0.999–1.001)	0.599		
Albumin < 35 g/L	0.864 (0.472–1.584)	0.637		
Surgical type (*n*/%)				
PD (*n*/%)	1.135 (0.641–2.009)	0.664		
Open operation (*n*/%)	1.133 (0.407–3.150)	0.811		
Estimated blood loss (ml)	1.000 (0.999–1.001)	0.571		
Transfusion (*n*/%)	1.630 (0.899–2.958)	0.108		
Cancer stage (*n*/%)		0.029		0.297
Cancer stage (I)	1.000		1.000	
Cancer stage (II)	2.618 (1.287–5.328)	0.008	1.852 (0.848–4.044)	0.122
Cancer stage (III)	2.110 (0.832–5.351)	0.116	1.493 (0.510–4.368)	0.464
Location (*n*/%)		0.020		0.075
Pancreas	1.000		1.000	
Ampulla of Vater	0.182 (0.025–1.318)	0.092	0.229 (0.031–1.697)	0.149
Duodenum	0.371 (0.115–1.200)	0.098	0.328 (0.075–1.448)	0.141
Common bile duct	0.109 (0.015–0.788)	0.028	0.145 (0.019–1.102)	0.062

*ALT* alanine transaminase, *ASA* American Society of Anesthesiologists, *BMI* body mass index, *CA-199* carbohydrate antigen 19-9, *CEA* carcinoembryonic antigen, *CHD* coronary heart disease, *CI* confidence interval, *CRP* C-reactive protein, *HR* hazard ratios, *PD* pancreaticoduodenectomy, *TB* total bilirubin

### Subgroup analysis

Given lesions located in different location may have their own prognostic factors and characteristic, we conducted an additional subgroup analysis according to different lesion location. As shown in Fig. S1a and b, in patients with pancreas cancer, *BMI* < 25.0 kg/m^2^ was significantly associated with OS (*P* = 0.025), while albumin < 35.0 g/L was not (*P* = 0.086). As depicted in Table S1, multivariable analysis further showed that *BMI* < 25.0 kg/m^2^ was an independent risk for OS with adjusted HR of 3.209 (95% *CI* = 0.985–10.451, *P* = 0.048). Additionally, there were no significant association of BMI and albumin with OS in patients with lesion in ampulla of Vater (*P* = 0.634; *P* = 0.371) (Fig. S2a and b), lesion in duodenum (*P* = 0.448; *P* = 0.813) (Fig. S3a and b), and lesion in common bile duct (*P* = 0.433; *P* = 0.433) (Fig. S4a and b).

## Discussion

This prospective cohort study was designed to explore whether preoperative BMI and serum albumin levels affected OS in patients undergoing PD for pancreatic cancer or periampullary cancer. We mainly demonstrate that preoperative *BMI* < 25.0 kg/m^2^ of patients undergoing PD had a decreased long-term OS independent of known clinicopathologic factors (adjusted *HR* = 3.516, 95% *CI* = 1.076–11.492, *P* = 0.037); however, preoperative albumin < 35 g/L was not identified the effects on long-term OS (*HR* = 0.864, 95% *CI* = 0.472–1.584, *P* = 0.637). We further performed subgroup analysis, and findings showed, in patients with pancreas lesion, *BMI* < 25.0 kg/m^2^ had significantly higher risks for OS compared to *BMI* ≥ 25.0 kg/m^2^ (adjusted *HR* = 3.209, 95% *CI* = 0.985–10.451, *P* = 0.048), while preoperative albumin < 35.0 g/L was not linked to OS (adjusted *HR* = 0.911, 95% *CI* = 0.450–1.844, *P* = 0.795). In patients with lesion in ampulla of Vater, duodenum, or common bile duct, there was no significant association of both BMI and albumin with OS.

The impact of BMI on OS in patients undergoing PD for pancreatic cancer has been controversial. On the one hand, a large sample study using a national clinical database found that obese patients (*BMI* ≥ 30 kg/m^2^) undergoing PD for pancreatic cancer had higher risks of postoperative 30-day mortality compared to control (*BMI* < 30 kg/m^2^) [24]. However, the authors presented that only 13% of the total number of obese patients were treated and thus had limited statistical power because of this constraint; some statistical analyses and inferences may be constrained. Additionally, in that study, the lack of long-term follow-up data led to an unknown impact of BMI on long-term OS. On the other hand, in consistent with our findings, a study by Tsai et al. stratified 795 patients undergoing PD for pancreatic cancer to obese (*BMI* > 30 kg/m^2^), overweight (*BMI* 25 to 30 kg/m^2^), and normal weight (*BMI* < 25 kg/m^2^) and showed that compared with normal weight, overweight and obese significantly increased by 32% and 28% risks of 5-year cancer-specific mortality respectively [23]. Notably, the authors excluded cases that had *BMI* ≤ 18.5 kg/m^2^. And it more strongly demonstrates that *BMI* < 25 kg/m^2^ was associated with decreased long-term OS in patients who underwent PD for pancreas cancer. Higher BMI with decreased risks of mortality may be explained by that higher BMI represents more nutritional reserve and enhanced inflammatory response to protect organism from damage [27, 38]. Therefore, supplementing nutrition and reducing inflammation for *BMI* < 25 kg/m^2^ of pancreatic cancer patients who are preparing for PD surgery need to be taken into account.

Studies which revealed the effects of BMI on OS in patients with lesion located in ampulla of Vater, duodenum, or common bile duct were fewer. In current study, there were no significant correlation between BMI and long-term OS. However, only 27, 34, and 39 cases with lesion in ampulla of Vater, duodenum, or common bile duct respectively were included in the analysis, and thus, the results may not be robust.

Albumin level is an easily available objective parameter and is also often observed in clinical practice. However, its association with OS in patients who underwent PD for pancreatic cancer remains controversial. In our study, there were no significant differences of OS between patients with albumin < 35 g/L and albumin ≥ 35 g/L. Similarly, a past large sample study found less than 35 g/L of serum albumin level was significantly associated with increased 30-day mortality for “borderline resectable type C” patients (defined as age ≥ 80, poor performance status, weight loss > 10%, pulmonary disease, recent myocardial infarction/angina, stroke history, and/or preoperative sepsis) who underwent PD, while association was not observed after adjusting confounding factors [25]. Unlike these findings, Coppola A et al. suggested that serum albumin levels may indirectly affect survival by affecting the ability of CA-199 to predict lymph node involvement in patients with pancreatic ductal adenocarcinoma [39]. The inconsistent results may be due to different study populations and serum albumin stratification criteria between the study by Coppola A et al. and ours. For example, the former included only patients with resectable pancreatic cancer with pathological stage less than 3, whereas our study included patients with pancreatic cancer who underwent pancreaticoduodenectomy, including patients with pathological stage 3. In addition, this study used 35 g/L as the cutoff value for serum albumin stratification, whereas 32 g/L was used in the study by Coppola A et al.

Studies that explored the effects of serum albumin on OS in patients with lesion located in ampulla of Vater, duodenum, or common bile duct were also limited. We did not observe the serum albumin < 35 g/L was significantly linked to long-term OS in patients with lesion located in ampulla of Vater, duodenum, or common bile duct. On the contrary, a retrospective study which included 79 patients who underwent PD for periampullary cancer considered albumin < 25 g/L as a risk factor for OS [26]. However, in that study, only 9 of total number of patients had albumin levels < 25 g/L, which limited the statistical power [26]. Thus, issues involving in the relationships between preoperative albumin level and OS in patients who underwent PD for periampullary lesions need further studies to address.

There were some limitations to current study. Firstly, this study ended follow-up on June 30, 2021, resulting in less than 1 year of follow-up for patients that received PD surgery after July 2020. Thus, we did not evaluate the effects of BMI and serum albumin on longer-term OS in patients who underwent PD. Additionally, as this was a retrospective study, current analysis was restrained by the data at hand. The number of patients with lesion in ampulla of Vater, duodenum, or common bile duct was less than 40; thus, it was insufficient. Further expanding the sample size to improve statistical performance is necessary.

## Conclusion

To sum up, firstly, *BMI* < 25.0 kg/m^2^, rather than albumin < 35 g/L, of patients undergoing PD had a decreased long-term OS independent of known clinicopathologic factors. Secondly, *BMI* < 25.0 kg/m^2^ of patients undergoing PD for lesion in pancreas but not ampulla of Vater, duodenum, or common bile duct was significantly linked to a reduced long-term OS. Thirdly, albumin < 35 g/L was not identified the effects on long-term OS in patients who underwent PD for lesions in pancreas, ampulla of Vater, duodenum, and common bile duct.

## Supplementary Information

**Additional file 1: Table S1.** Univariate and multivariate analysis of factors associated with overall survival in patients undergoing PD for lesion in pancreas (*n* =229). **Fig. S1.** a. Kaplan-Meier survival curves for overall cohort in patients undergoing PD for lesion in pancreas according to BMI (<25.0 Kg/m^2^ VS. ≥25.0 Kg/m^2^) (Note: Group 1, BMI<25 Kg/m^2^; Group 2, ≥25 Kg/m^2^). b. Kaplan-Meier survival curves for overall cohort in patients undergoing PD for lesion in pancreas according to albumin (<35.0 g/L VS. ≥35.0 g/L) (Note: Group I, serum albumin <35 g/L; Group II, serum albumin ≥35 g/L). **Fig. S2.** a. Kaplan-Meier survival curves for overall cohort in patients undergoing PD for lesion in ampulla of Vater according to BMI (<25.0 Kg/m^2^ VS. ≥25.0 Kg/m^2^) (Note: Group 1, BMI<25 Kg/m^2^; Group 2, ≥25 Kg/m^2^). b. Kaplan-Meier survival curves for overall cohort in patients undergoing PD for lesion in ampulla of Vater according to albumin (<35.0 g/L VS. ≥35.0 g/L) (Note: Group I, serum albumin <35 g/L; Group II, serum albumin ≥35 g/L). **Fig. S3.** a. Kaplan-Meier survival curves for overall cohort in patients undergoing PD for lesion in duodenum according to BMI (<25.0 Kg/m^2^ VS. ≥25.0 Kg/m^2^) (Note: Group 1, BMI<25 Kg/m^2^; Group 2, ≥25 Kg/m^2^). b. Kaplan-Meier survival curves for overall cohort in patients undergoing PD for lesion in duodenum according to albumin (<35.0 g/L VS. ≥35.0 g/L) (Note: Group I, serum albumin <35 g/L; Group II, serum albumin ≥35 g/L). **Fig. S4.** a. Kaplan-Meier survival curves for overall cohort in patients undergoing PD for lesion in common bile duct according to BMI (<25.0 Kg/m^2^ VS. ≥25.0 Kg/m^2^) (Note: Group 1, BMI<25 Kg/m^2^; Group 2, ≥25 Kg/m^2^). b. Kaplan-Meier survival curves for overall cohort in patients undergoing PD for lesion in common bile duct according to albumin (<35.0 g/L VS. ≥35.0 g/L) (Note: Group I, serum albumin <35 g/L; Group II, serum albumin ≥35 g/L).

## Data Availability

All data and materials support the published claims and comply with field standards.
