# Supplementary material for: Effects of body mass index and serum albumin on overall survival in patients with cancer undergoing pancreaticoduodenectomy: a single-center retrospective cohort study
Source: World J Surg Oncol. 2022 Jul 1;20:221. doi: 10.1186/s12957-022-02678-z (PMC9248124; doi:10.1186/s12957-022-02678-z)
Supplement: Supplementary file 1 — Additional file 1: Table S1. Univariate and multivariate analysis of factors associated with overall survival in patients undergoing PD for lesion in pancreas (n =229). Fig. S1. a. Kaplan-Meier survival curves for overall cohort in patients undergoing PD for lesion in pancreas according to BMI (<25.0 Kg/m2 VS. ≥25.0 Kg/m2) (Note: Group 1, BMI<25 Kg/m2; Group 2, ≥25 Kg/m2). b. Kaplan-Meier survival curves for overall cohort in patients undergoing PD for lesion in pancreas according to albumin (<35.0 g/L VS. ≥35.0 g/L) (Note: Group I, serum albumin <35 g/L; Group II, serum albumin ≥35 g/L). Fig. S2. a. Kaplan-Meier survival curves for overall cohort in patients undergoing PD for lesion in ampulla of Vater according to BMI (<25.0 Kg/m2 VS. ≥25.0 Kg/m2) (Note: Group 1, BMI<25 Kg/m2; Group 2, ≥25 Kg/m2). b. Kaplan-Meier survival curves for overall cohort in patients undergoing PD for lesion in ampulla of Vater according to albumin (<35.0 g/L VS. ≥35.0 g/L) (Note: Group I, serum albumin <35 g/L; Group II, serum albumin ≥35 g/L). Fig. S3. a. Kaplan-Meier survival curves for overall cohort in patients undergoing PD for lesion in duodenum according to BMI (<25.0 Kg/m2 VS. ≥25.0 Kg/m2) (Note: Group 1, BMI<25 Kg/m2; Group 2, ≥25 Kg/m2). b. Kaplan-Meier survival curves for overall cohort in patients undergoing PD for lesion in duodenum according to albumin (<35.0 g/L VS. ≥35.0 g/L) (Note: Group I, serum albumin <35 g/L; Group II, serum albumin ≥35 g/L). Fig. S4. a. Kaplan-Meier survival curves for overall cohort in patients undergoing PD for lesion in common bile duct according to BMI (<25.0 Kg/m2 VS. ≥25.0 Kg/m2) (Note: Group 1, BMI<25 Kg/m2; Group 2, ≥25 Kg/m2). b. Kaplan-Meier survival curves for overall cohort in patients undergoing PD for lesion in common bile duct according to albumin (<35.0 g/L VS. ≥35.0 g/L) (Note: Group I, serum albumin <35 g/L; Group II, serum albumin ≥35 g/L). [file 12957_2022_2678_MOESM1_ESM.docx]

**Table S1 Univariate and multivariate analysis of factors associated with overall survival in patients undergoing PD for lesion in pancreas (n=229)**

| **Variables** | **Unadjusted HR (95% CI)** | ***P* value** | **Adjusted HR (95% CI)** | ***P-*value** |
| --- | --- | --- | --- | --- |
| Age (≥ 65 years) (n/%) | 2.771(1.430-5.369) | 0.003 | 2.639(1.317-5.288) | 0.006 |
| Male (n/%) | 0.901(0.500-1.623) | 0.729 |  |  |
| Smoking history (n/%) | 0.852(0.396-1.830) | 0.681 |  |  |
| Drinking history (n/%) | 2.064(0.638-6.675) | 0.226 |  |  |
| Diabetes (n/%) | 0.910(0.461-1.796) | 0.785 |  |  |
| Hypertension (n/%) | 1.082(0.588-1.993) | 0.800 |  |  |
| CHD (n/%) | 0.048(0.000-151.099) | 0.459 |  |  |
| ASA Class (II) | 1.291(0.176-9.456) | 0.802 |  |  |
| ASA Class (III) | 2.375(0.308-18.292) | 0.406 |  |  |
| BMI <25.0 Kg/m^2^ | 3.046(1.091-8.506) | 0.034 | 3.209(0.985-10.451) | 0.048 |
| CA-199 (U/ml) | 1.000(1.000-1.001) | 0.321 |  |  |
| CEA(ng/ml) | 0.944(0.871-1.023) | 0.162 |  |  |
| CRP (mg/L) | 1.011(1.002-1.020) | 0.019 | 1.010(1.000-1.020) | 0.042 |
| HbA1c (n/%) | 0.897(0.699-1.152) | 0.395 |  |  |
| ALT (U/L) | 1.000(0.999-1.002) | 0.608 |  |  |
| Bilirubin (μmol/l) | 1.001(0.999-1.002) | 0.445 |  |  |
| Albumin<35 g/L | 0.578(0.307-1.089) | 0.090 | 0.911(0.450-1.844) | 0.795 |
| PD | 1.403(0.754-2.610) | 0.285 |  |  |
| Open operation (n/%) | 0.929(0.288-2.999) | 0.902 |  |  |
| Estimated blood loss (ml) | 1.000(0.999-1.001) | 0.863 |  |  |
| Transfusion (n/%) | 1.496(0.795-2.814) | 0.212 |  |  |
| Cancer stage (n/%) | -- | 0.438 |  |  |
| Cancer Stage(I) | 1.000 | -- |  |  |
| Cancer stage(II) | 1.663(0.760-3.638) | 0.203 |  |  |
| Cancer Stage(III) | 1.390(0.521-3.704) | 0.511 |  |  |

**Note:** ALT, alanine transaminase; ASA, American Society of Anesthesiologists; BMI, body mass index; CA-199, carbohydrate antigen 19-9; CEA, carcinoembryonic antigen; CHD, Coronary Heart Disease; CI, Confidence Interval; CRP, C-reactive protein; HR, Hazard Ratios; PD, pancreaticoduodenectomy; TB, total bilirubin


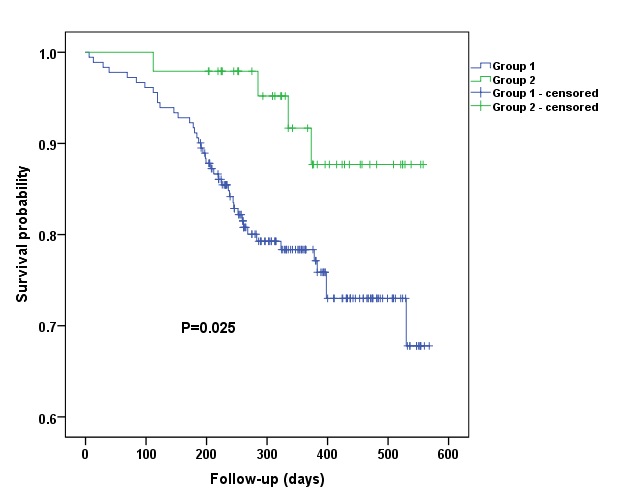


**Fig. S1a Kaplan-Meier survival curves for overall cohort in patients undergoing PD for lesion in pancreas according to BMI (<25.0 Kg/m^2^ VS. ≥25.0 Kg/m^2^)**

**(Note:** Group 1, BMI<25 Kg/m^2^; Group 2, ≥25 Kg/m^2^)


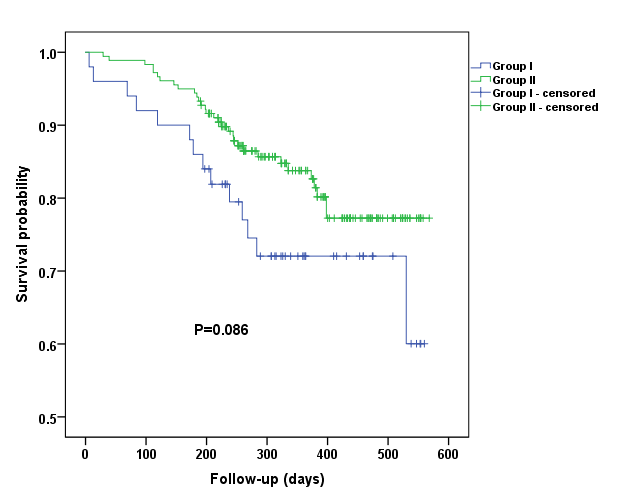


**Fig. S1b Kaplan-Meier survival curves for overall cohort in patients undergoing PD for lesion in pancreas according to albumin (<35.0 g/L VS. ≥35.0 g/L)**

**(Note:** Group I, serum albumin <35 g/L; Group II, serum albumin ≥35 g/L)


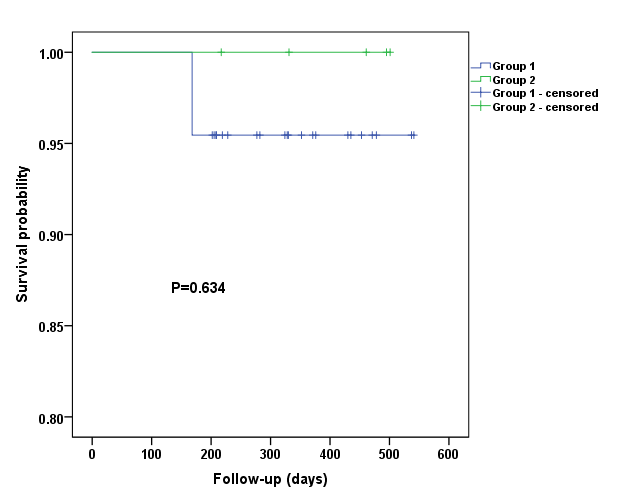


**Fig. S2a Kaplan-Meier survival curves for overall cohort in patients undergoing PD for lesion in ampulla of Vater according to BMI (<25.0 Kg/m^2^ VS. ≥25.0 Kg/m^2^)**

**(Note:** Group 1, BMI<25 Kg/m^2^; Group 2, ≥25 Kg/m^2^)


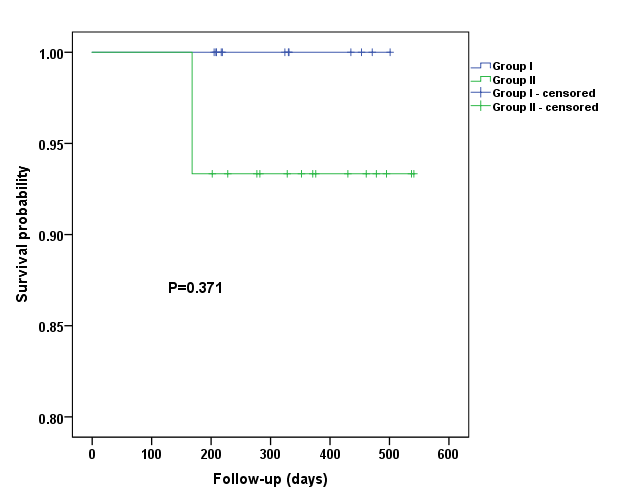


**Fig. S2b Kaplan-Meier survival curves for overall cohort in patients undergoing PD for lesion in ampulla of Vater according to albumin (<35.0 g/L VS. ≥35.0 g/L)**

**(Note:** Group I, serum albumin <35 g/L; Group II, serum albumin ≥35 g/L)


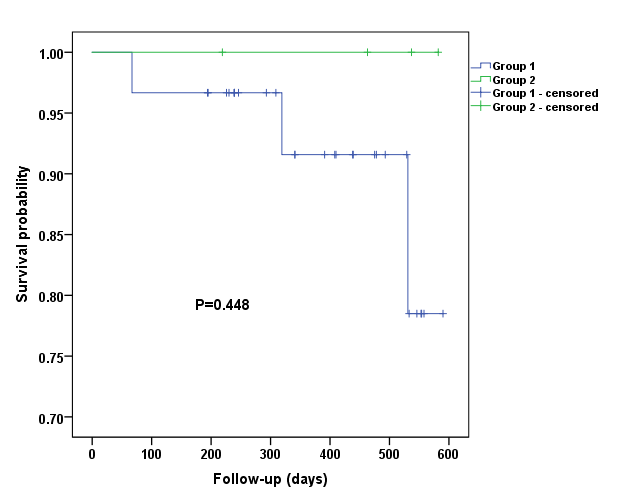


**Fig. S3a Kaplan-Meier survival curves for overall cohort in patients undergoing PD for lesion in duodenum according to BMI (<25.0 Kg/m^2^ VS. ≥25.0 Kg/m^2^)**

**(Note:** Group 1, BMI<25 Kg/m^2^; Group 2, ≥25 Kg/m^2^)


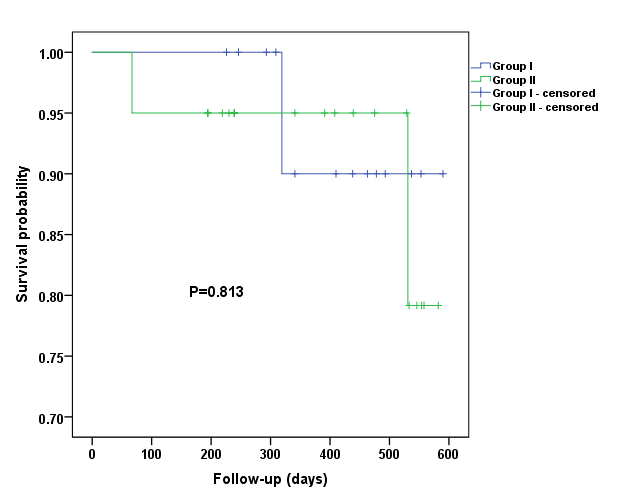


**Fig. S3b Kaplan-Meier survival curves for overall cohort in patients undergoing PD for lesion in duodenum according to albumin (<35.0 g/L VS. ≥35.0 g/L)**

**(Note:** Group I, serum albumin <35 g/L; Group II, serum albumin ≥35 g/L)


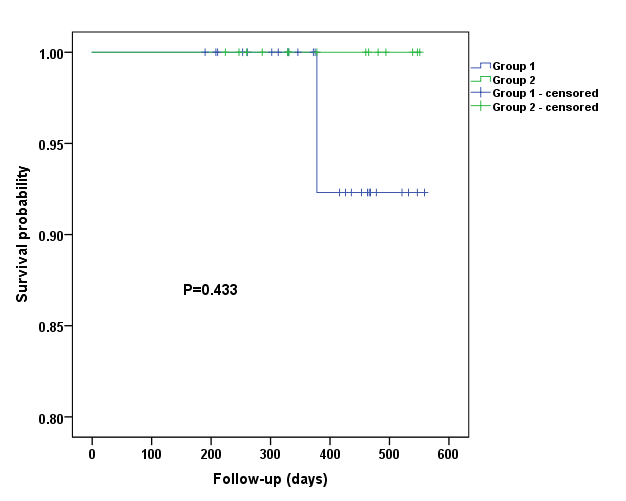


**Fig. S4a Kaplan-Meier survival curves for overall cohort in patients undergoing PD for lesion in common bile duct according to BMI (<25.0 Kg/m^2^ VS. ≥25.0 Kg/m^2^)**

**(Note:** Group 1, BMI<25 Kg/m^2^; Group 2, ≥25 Kg/m^2^)


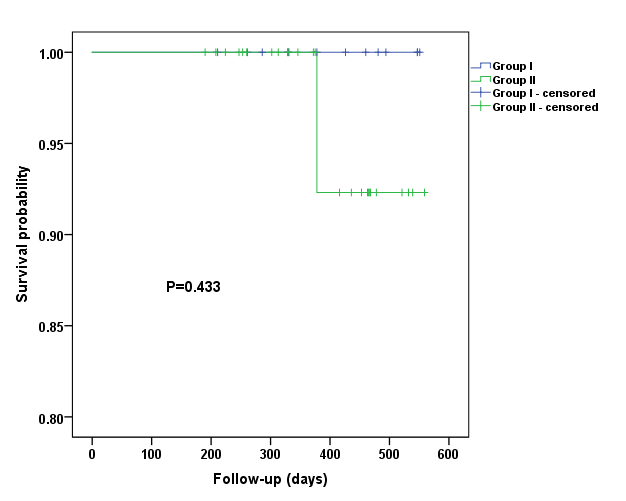


**Fig. S4b Kaplan-Meier survival curves for overall cohort in patients undergoing PD for lesion in common bile duct according to albumin (<35.0 g/L VS. ≥35.0 g/L)**

**(Note:** Group I, serum albumin <35 g/L; Group II, serum albumin ≥35 g/L)
